# Supplementary figures and images for: Fasciclin 2 functions as an expression-level switch on EGFR to control organ shape and size in Drosophila
Source: PLoS One. 2024 Dec 20;19(12):e0309891. doi: 10.1371/journal.pone.0309891 (PMC11661588; doi:10.1371/journal.pone.0309891)

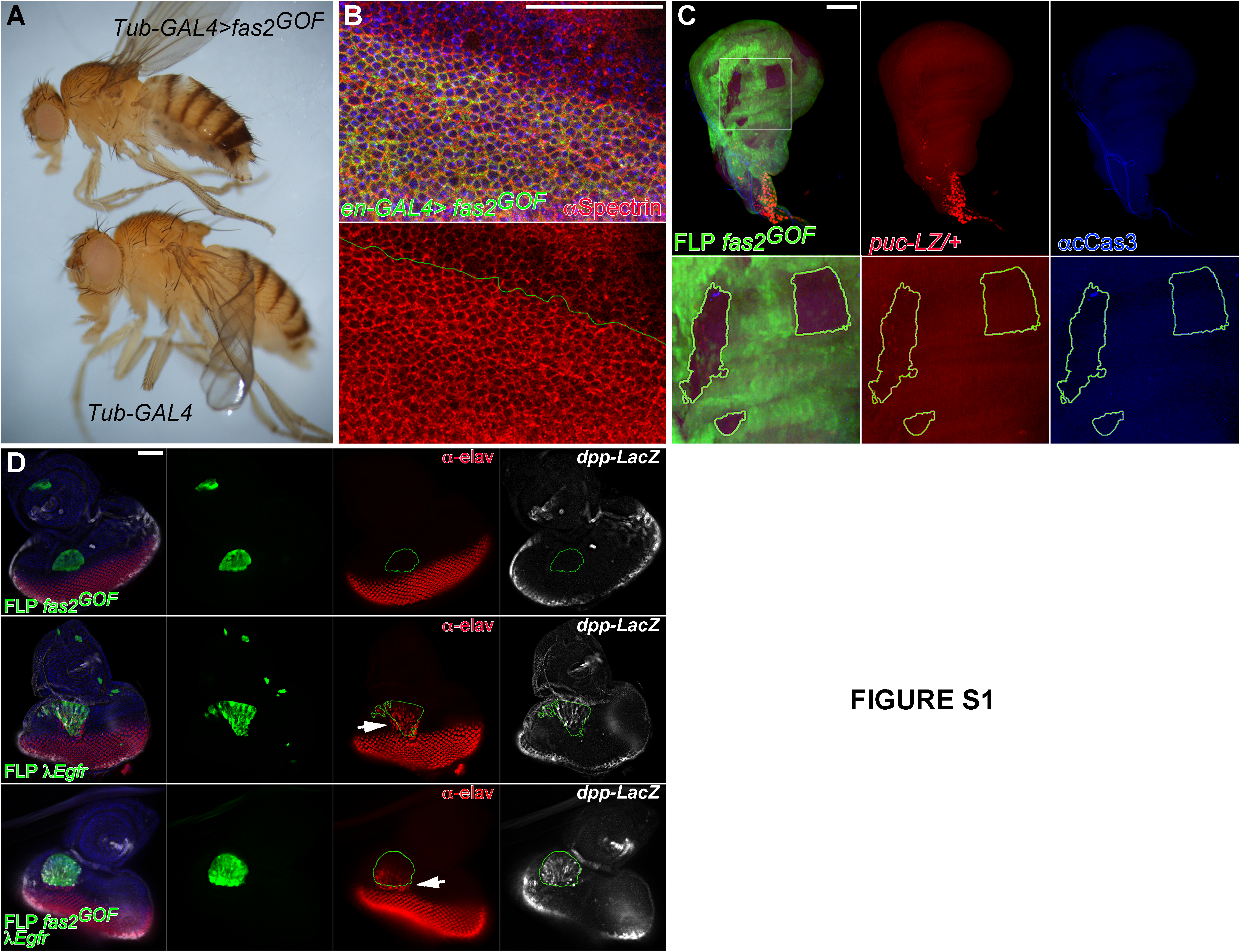

Supplement: S1 Fig — (A) Over-expression of Fas2TRM (UAS-fas2TRM) under the control of the Tub-GAL4/+ driver (Tub-GAL4>fas2GOF) caused a general reduction of body size. Compare the two females, with over-expression of Fas2TRM (top) and a normal CyO sibling (Tub-GAL4) control (bottom). (B) A/P compartment border in an en-GAL4 UAS-CD8GFP/UAS-fas2TRM UAS-fas2GPI pupal wing. The expression of Fas2GPI and Fas2TRM in the Posterior compartment of the wing (labelled with GFP) did not modify the size or shape of the cells. Bar: 50 μm. (C) FLP-OUT fas2 GOF clones in a puc-LacZ/+ genetic background did not show increased JNK activity nor apoptosis. At left, a wing disc with several Fas2 GOF clones (labeled with GFP) which cover most of the disc surface. The lack of ectopic expression from the reporter puc-LacZ reveals that the JNK pathway was not de-repressed in these clones. Staining with anti-cleaved Caspase 3 (Blue channel) did not reveal obvious signs of apoptosis in the wing discs either. Lower panels, note the presence of just three cells expressing cleaved Caspase 3 in one of the normal regions engulfed by the Fas2 GOF clones. Bar: 50 μm. (D) FLP-OUT y w hs-FLP; ActinFRTy+FRT-GAL4 UAS-GFP/UAS-fas2TRM UAS-fas2GPI; UAS-λEgfr/dpp-LacZ clones (middle panels) display the same phenotype than FLP-OUT y w hs-FLP; ActinFRTy+FRT-GAL4 UAS-GFP/+; UAS-λEgfr/dpp-LacZ clones (bottom panels). In both types of clones there is precocious non-cell autonomous retinal differentiation (arrows) and ectopic dpp expression inside the clone. Bar: 50 μm. (TIF) [file pone.0309891.s001.tif]

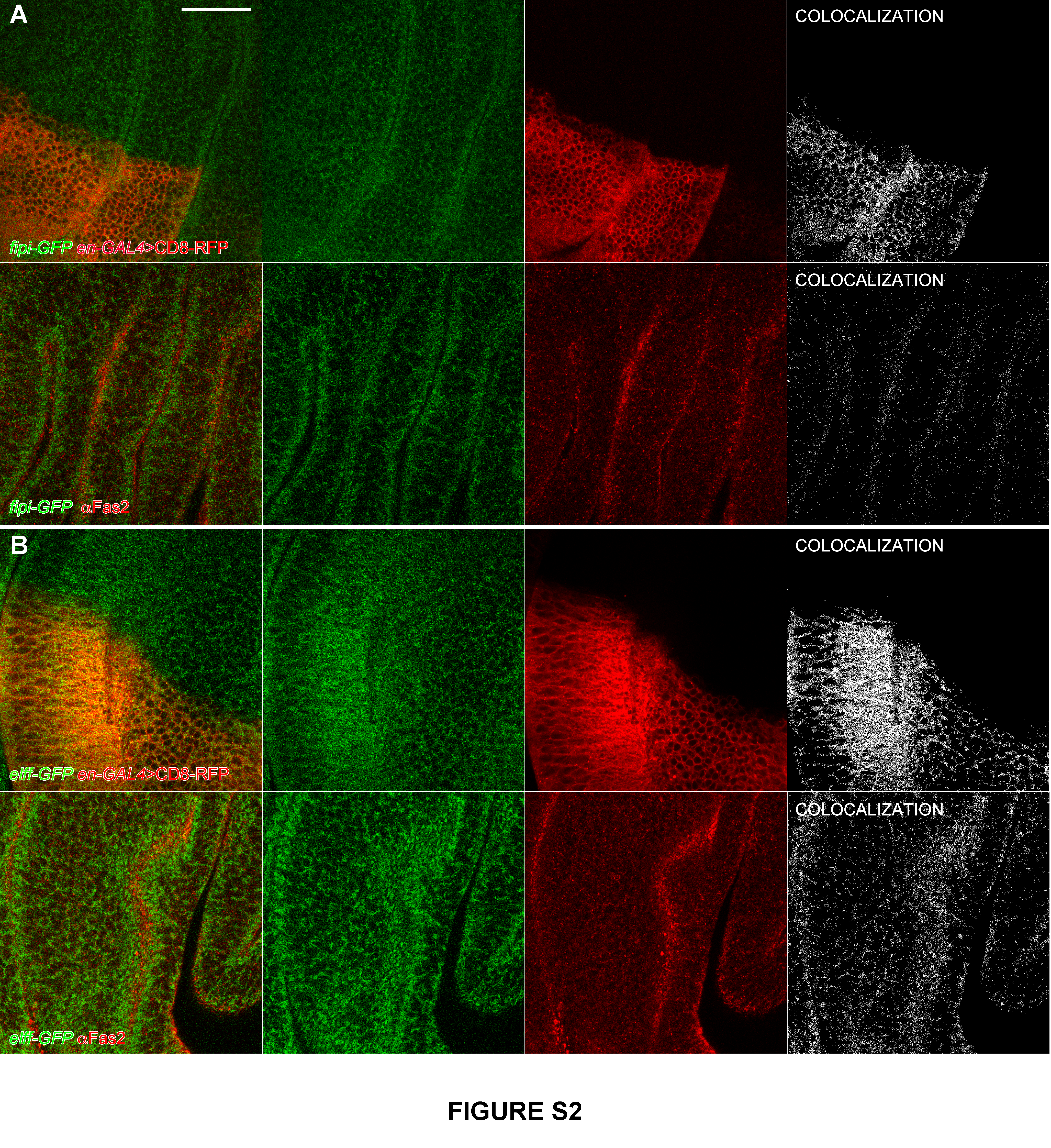

Supplement: S2 Fig — (A) Top: the protein trap line fipi-EGFP-FLAG (RRID: BDSC_60532) shows protein colocalization with the membrane marker CD8-RFP. 3rd instar wing imaginal disc stained with anti-Flag (green) and expressing UAS-CD8-RFP under the control of the en-GAL4/+ driver. ImageJ Colocalization and Colocalization Finder plugins, ratio: 50.0; threshold red: 100.0; threshold green: 100.0; Pearson’s correlation: + 0,349. Bottom: the expression of fipi-EGFP-FLAG (RRID:BDSC_60532) (anti-Flag, green) partially colocalizes with Fas2 (Mab 1D4, red) in the wing imaginal disc during late 3rd instar larva. Expression of both proteins in different domains is better seen in the lateral views of cells at the imaginal disc folds. Note that the partial colocalization corresponds to the interface between Fas2 (red) and Fipi (green). ImageJ Colocalization and Colocalization Finder plugins, ratio: 50.0; threshold red: 100.0; threshold green: 100.0; Pearson’s correlation: + 0,332. Bar: 50 μm. (B) Top: the protein trap line elff-EGFP-FLAG (RRID:BDSC_60531) also shows colocalization with the membrane marker CD8-RFP. 3rd instar wing imaginal disc stained with anti-Flag (green) and expressing UAS-CD8-RFP under the control of the en-GAL/+4 driver. ImageJ Colocalization and Colocalization Finder plugins, ratio: 50.0; threshold red: 100.0; threshold green: 100.0; Pearson’s correlation: + 0,419. Bottom: elff-EGFP-FLAG (RRID:BDSC_60531) (anti-Flag, green) partially colocalizes with Fas2 (Mab 1D4, red) during late 3rd instar larva. The panel shows the cell profiles at folds near the prospective wing pouch. Note the colocalization at the apposition of red (Fas2) and green (Elff) signals. ImageJ Colocalization and Colocalization Finder plugins, ratio: 50.0; threshold red: 100.0; threshold green: 100.0; Pearson’s correlation: + 0,392. (TIF) [file pone.0309891.s002.tif]

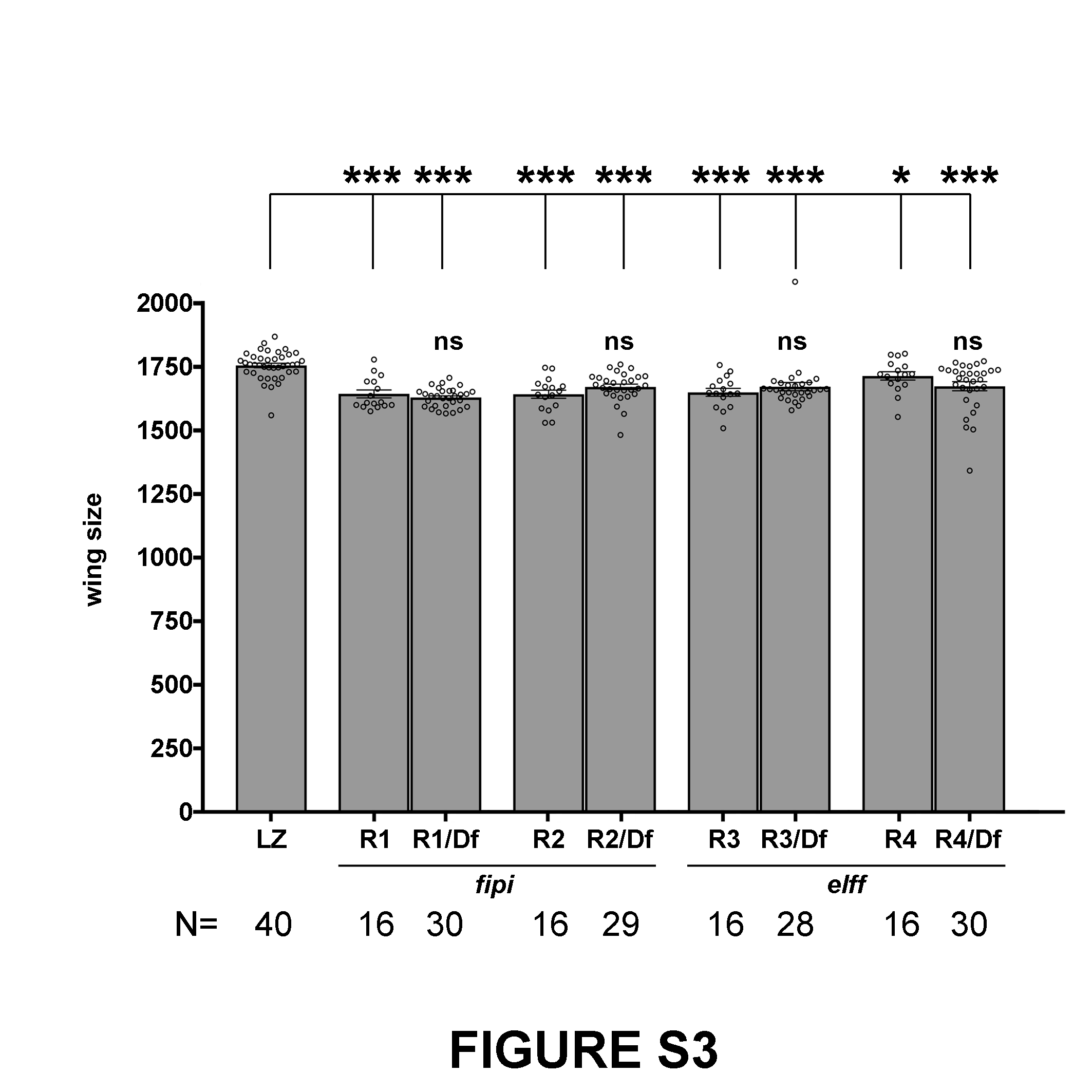

Supplement: S3 Fig — The results for fipi and elff RNAi inhibition presented in Fig 5C are here compared with the same combinations over a deficiency that removes the corresponding endogenous gene. The phenotype caused by UAS-fipi or UAS-elff RNAi expression driven by MS1096/+ is not significantly enhanced by reducing 50% the dose of the corresponding gene using Df(2L)BSC225, fipi−(RRID:BDSC_9702) and Df(2L)Exel7008, elff−(RRID:BDSC_7780). The results show that these RNAis block most fipi and elff function causing at least a strong hypomorphic condition. Wing size is area in μm2/103. (TIF) [file pone.0309891.s003.tif]

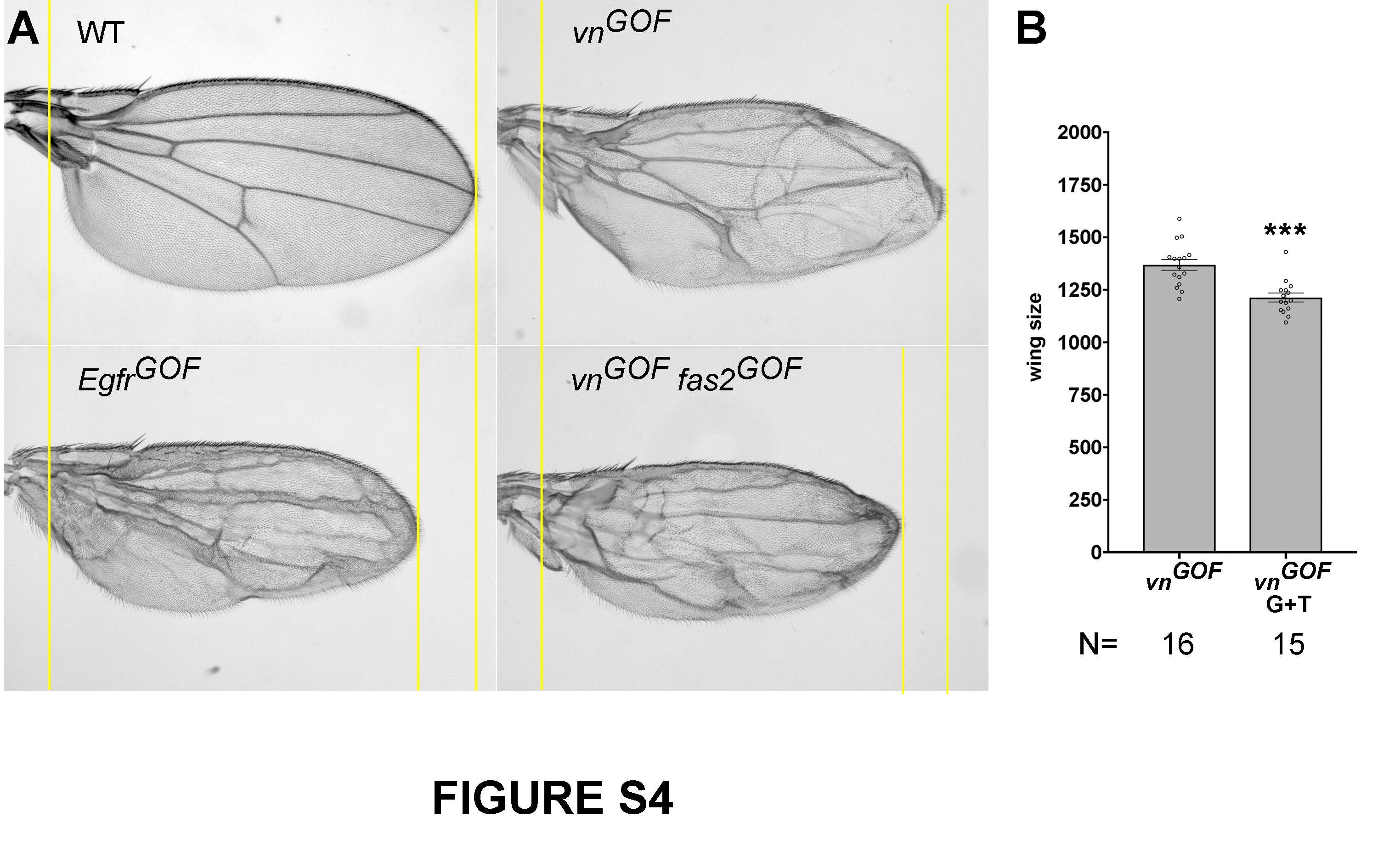

Supplement: S4 Fig — (A) The gain of function for EGFR (EgfrGOF, MS1096-GAL4/+; Egfrλtop/+; bottom left) during imaginal wing disc growth produces adult wings smaller than normal (WT, MS1096/+, top left), and with a pronounced differentiation of extra-vein territory. A similar situation is attained by the over-expression of the EGF-like ligand Vein (vnGOF; top right). Both, the reduction in wing size and the extra-vein phenotype caused by the over- expression of UAS-vn (under the control of the MS1096-GAL4/+ driver, vnGOF) is enhanced by the simultaneous over-expression of fas2 (vnGOF fas2GOF, MS1096/+; UAS-vn/UAS- fas2GPI UAS-fas2TRM; bottom right). (B) Quantification of wing area in over-expression conditions for Vn (vnGOF) and Vn plus Fas2GPI Fas2TRM (vnGOF G+T) under the control of the MS1096-GAL4/+ driver. All pictures heterozygous MS1096-GAL4/+ females raised at 25°C. Wing size is area in μm2/103. (TIF) [file pone.0309891.s004.tif]

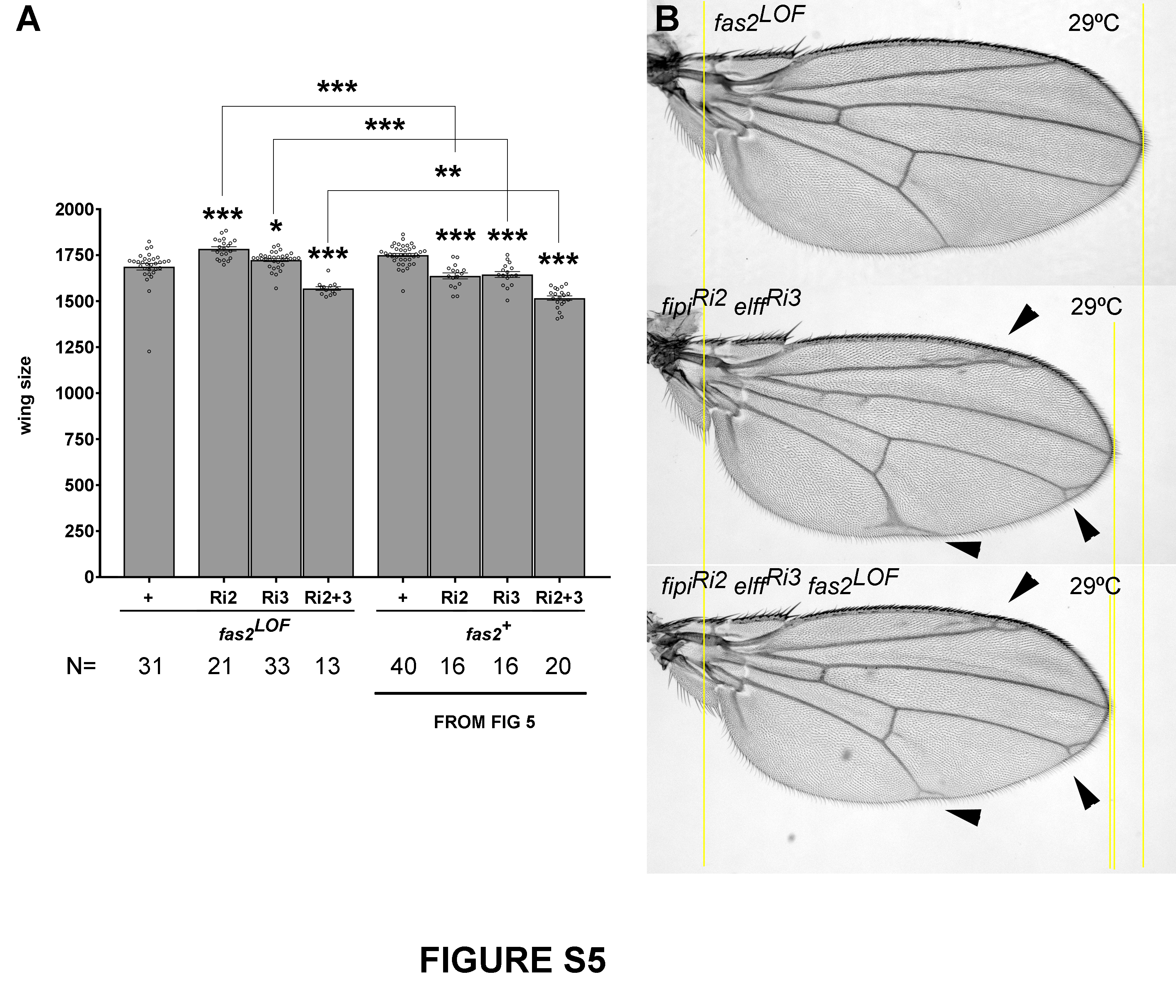

Supplement: S5 Fig — (A) Inhibition of fas2 (RNAi RRID: BDSC_28990) in combination with fipi (RNAi RRID:BDSC_42589, Ri2) or elff (RNAi RRID:VDRC_32576, Ri3) in MS1096-GAL4/+ females causes a normalized adult wing size. The double inhibition of fipi and elff expression in the fas2 LOF background shows a phenotype slightly suppressed. The combinations fipi elff are the same as in Fig 5C. All individuals are heterozygous MS1096-GAL4/+ females raised at 25°C. Wing size is area in μm2/103. (B) The inhibition of fas2 by the expression of RNAi (RRID:BDSC_28990) in MS1096-GAL4/+ females (fas2LOF) raised at 29°C does not produce alterations in the wing vein pattern (with the exception of missing cross-veins in some individuals). Simultaneous inhibition of fipi and elff (RNAi RRID:BDSC_42589 and RNAi RRID:VDRC_32576) in MS1096-GAL4/+ females (fipiRi2 elffRi3) raised at 29°C causes the differentiation of extra-vein tissue (arrowheads), while the simultaneous inhibition of fas2 fipi and elff expression (fipiRi2 elffRi3 fas2LOF) does not eliminate the formation of extra-veins (arrowheads). All pictures heterozygous MS1096-GAL4/+ females raised at 29°C. (TIF) [file pone.0309891.s005.tif]
