# Supplementary material for: Fasciclin 2 functions as an expression-level switch on EGFR to control organ shape and size in Drosophila
Source: PLoS One. 2024 Dec 20;19(12):e0309891. doi: 10.1371/journal.pone.0309891 (PMC11661588; doi:10.1371/journal.pone.0309891)
Supplement: S1 File — Strains used for MARCM and coupled-MARCM analysis. (DOCX) [file pone.0309891.s006.docx]

Strains used for MARCM and coupled-MARCM analysis:

*y QS13F FRT19A/FM7-GFP; QF-ET40 QUAS-mtdTomato/CyO*

*y QS13F FRT19A; UAS-fas2^GPI^ UAS-fas2^TRM^*

*w hsp70-flp Tub-GAL80 FRT19A; Tub-GAL4 UAS-GFP/TM6B*

*y ey-flp Tub-GAL80 FRT19A; Tub-Gal4 UAS-GFP/TM6B*

Strains for FLP-OUT clone analysis:

*y ey-flp; Act5C-FRTy^+^FRT-GAL4 UAS-GFP/CyO*

*y w hsp70-flp; Act5C-FRTy^+^FRT-GAL4 UAS-GFP/CyO*

*y w hsp70-flp; Act5C-FRTy^+^FRT-GAL4 UAS-GFP/CyO; Tub-miniCic::scarlet*

*y w hsp70-flp; Act5C-FRTy^+^FRT-GAL4 UAS-GFP*; *puc-LacZ^E69^/TM3*

*y w hsp70-flp; Act5C-FRTy^+^FRT-GAL4 UAS-GFP; Act5C-FRT-polyA-FRT-LacZ.*

Strains used for time controlled *UAS*-mediated expression:

*w; TubGAL80^ts20^/CyO; Tub-GAL4 UAS-GFP/TM6B*

*MS1096-GAL4/FM6; Tub-GAL80^ts20^/CyO*

*w; Tub-GAL80^ts20^/CyO; hsp-GAL4*
